# Molecular markers-based DNA fingerprinting coupled with morphological diversity analysis for prediction of heterotic grouping in sunflower (*Helianthus annuus* L.)

**DOI:** 10.3389/fpls.2022.916845

**Published:** 2022-07-28

**Authors:** Danish Ibrar, Shahbaz Khan, Talat Mahmood, Ali Bakhsh, Irum Aziz, Afroz Rais, Rafiq Ahmad, Saqib Bashir, Muhammad Nawaz, Nabila Rashid, Sohail Irshad, Saqer S. Alotaibi, Helena Dvorackova, Jan Dvoracek, Zuhair Hasnain

**Affiliations:** ^1^National Agricultural Research Centre, Islamabad, Pakistan; ^2^Department of Plant Breeding and Genetics, Pir Mehr Ali Shah Arid Agriculture University Rawalpindi, Rawalpindi, Pakistan; ^3^Department of Plant Breeding and Genetics, Ghazi University, Dera Ghazi Khan, Pakistan; ^4^Department of Botany, Sardar Bahadur Khan Women's University, Quetta, Pakistan; ^5^Barani Agriculture Research Institute, Chakwal, Pakistan; ^6^Department of Soil and Environmental Science, Ghazi University, Dera Ghazi Khan, Pakistan; ^7^Department of Agricultural Engineering, Khwaja Fareed University of Engineering and Information Technology, Rahim Yar Khan, Pakistan; ^8^Department of Botany, University of Agriculture, Faisalabad, Pakistan; ^9^Department of Agronomy, MNS-University of Agriculture, Multan, Pakistan; ^10^Department of Biotechnology, College of Science, Taif University, Taif, Saudi Arabia; ^11^Department of Agrochemistry, Soil Science, Microbiology and Plant Nutrition, Faculty of AgriSciences, Mendel University in Brno, Brno, Czechia; ^12^Pedologiejh, spol s.r.o, Brno, Czechia; ^13^Department of Agronomy, Pir Mehr Ali Shah Arid Agriculture University Rawalpindi, Rawalpindi, Pakistan

**Keywords:** multivariate, sunflower, morphological, SSR, heterosis, yield

## Abstract

Cultivated sunflower holds a very narrow genetic base and the efficient utilization of available genetic diversity is very important for a successful breeding program. In the present study, 109 sunflower genotypes were assessed for diversity paneling through a combined approach of morphological and molecular markers analysis. Morphological parameters including days to flower initiation, days to flower completion, plant height, stem curvature, number of leaves per plant, leaf area, head diameter, hundred seed weight, and seed yield per plant were studied. Simple sequence repeats (40 DNA markers) were deployed for diversity profiling. Data were analyzed by both univariate and multivariate statistics. SD and coefficient of variation confirm the presence of significant amounts of genetic variation for all the morphological parameters. Cluster Analysis and Principal Component Analysis further confirm the presence of distinct grouping patterns in the studied material. Cluster analysis of both morphological and molecular analysis revealed that restorer lines tend to group separately from A, B, and open-pollinated lines. Further grouping, at the sub-cluster level, revealed six distinct sub-clusters in each of the two major clusters. In total, 12 genotypes, 6 CMS lines (CMS-HAP-12, CMS-HAP-54, CMS-HAP-56, CMS-HAP-99, CMS-HAP-111, and CMS-HAP-112) and 6 restorer lines (RHP-38, RHP-41, RHP-53, RHP-68, RHP-69, and RHP-71) could be used as potential parents for hybrid development. As genotypes of similar genetic backgrounds tend to group closer, it is deduced that one genotype with the highest seed yield per plant could be used for further hybrid breeding programs in sunflowers.

## Introduction

Sunflower (*Helianthus annuus* L.) is one of the two domesticated species of the family Asteracea, and the genus Helianthus which has more than 70 plant species (Lagiso et al., 2021). Sunflower is frequently cultivated to get premium quality vegetable oil from its seeds that have excellent nutritional qualities and health benefits (Dudhe et al., 2020). Sunflower oil is a triglyceride and contains palmitic acid 5%, stearic acid 6%, oliec acid 30%, and linoleic acid 59%, and is also free from harmful compounds such as erucic acid and glucosinolates (Aslam et al., 2021). In addition to 40–45% of oil content (Skoric, 1992; Leon et al., 2003), sunflower seeds also contain protein (23%) (Tahir et al., 2002), carbohydrates (30%), and ash (4%) (Hasan et al., 2020). Furthermore, sunflower oil is light in color, bland in flavor, lacks linolenic acid, and has a high percentage of unsaturated fatty acids with a high-smoke point. Because of these characteristics, sunflower oil is believed to be a premium quality vegetable oil.

The popularity of sunflower cultivation is due to wider adaptability, self-incompatibility, high yield, and uniformity along with two growing seasons in a calendar year (Sujatha and Nandini, 2002a; Kaya and Atakisi, 2004). With the intention to increase the productivity of a plant, a breeder can opt for two strategies: one is to develop a plant variety that can withstand elevated levels of biotic and abiotic stresses and the second one is to breed high-performing hybrids by crossing superior inbred lines (Falconer and Mackay, 1996). In general, it was observed that F_1_s obtained from distantly related inbred lines are more productive and adaptable than those closely related (Sujatha and Nandini, 2002b). Knowledge of the genetic components is a prerequisite for a plant breeder for improving the crop and yield potential that can only be enhanced by manipulating these yields and contributing to the genetically controlled characteristics (Arshad et al., 2007).

Phenotypic and genotypic variabilities are the most vital attributes to distinguish the efficiency of various oil and yield-linked traits correlation (Resende and de Duarte, 2007). Variation in a plant/line can be assessed morphologically and then their phenotypic and genotypic coefficients of variation are computed to assess the presence of genetic variability among different agronomic characters (Sujatha and Nandini, 2002a; Al-Mehemdi et al., 2020). Assessing the genetic diversity by agro-morphological characters is often time-consuming and laborious, especially when no prior knowledge of the variability is present (Lochner, 2011; Ikram et al., 2020). It is not a reliable technique as the morphological characters are highly influenced by the environment. Yield is a complex quantitative trait as it is dependent on other characters that may be misleading while making the selection process less efficient (Dewey and Lu, 1959; Ahmad et al., 2021), particularly, in the field crops such as sunflower, which is highly cross-pollinating and heterozygous and envisages enormous variations in successive generations (Arshad et al., 2007; Benchasri et al., 2020). The use of DNA markers can solve this problem as this technique is more reliable because it is not influenced by the environment. They are abundant as compared with the morphological and biochemical markers, and show both dominant (RAPD and AFLP) and co-dominant (RFLP and SSRs) inheritance.

From the different types of molecular-based DNA markers being utilized for similarity/distance estimation studies, SSRs have become the markers of choice due to their informative nature, easy development protocols, and cost-effective assays (Singh et al., 2020). The ability of the SSR markers to highlight the DNA sequences responsible for coding the proteins involved in many important agronomic characteristics has been demonstrated previously by many researchers in sunflower (Zeinalzadeh-Tabrizi et al., 2018) and also in other important agricultural crops such as soybean (Kumar et al., 2022), sugar beet (Singh et al., 2020), and other agricultural crops (Noor et al., 2021). Genetic diversity between parents of the crosses indicates differences in gene frequency, which can be exploited to improve inbred or parental lines (Ram et al., 2018). Information on genetic diversity could be used to predict the performance of inbred lines in a cross, as it is generally believed that a larger genetic distance among parental lines will lead to the development of productive hybrids (Avin et al., 2013). Hence, the genetic diversity information enables the breeder to focus on more promising cross combinations and, therefore, will accelerate the efficiency of a breeding program (Lochner, 2011).

Genetic divergence estimation has been proved useful in predicting the hybrid performance and drawing of strategies/methodologies for breeding and improvement of hybrids/cultivars. Therefore, objective of the present study was to explore the heterotic grouping among sunflower lines, so that this identified grouping pattern can be utilized efficiently in future sunflower-breeding programs. Prediction of heterotic groups was based on the genetic diversity assessed at the morphological level through nine plant attributes, i.e., plant height, stem curvature, number of leaves per plant, leaf area, time taken to flower initiation, flower completion, head diameter, seed yield per plot and 100-seed weight, coupled with genetic diversity analyzed at the molecular level through SSR/microsatellite markers.

## Materials and methods

### Experimental details

An experiment was conducted in the field area of the National Agricultural Research Center (NARC), Islamabad, which is situated on latitude 33.6641°N, and longitude 73.1276°E. In total, one hundred and nine diverse sunflower genotypes (Supplementary Table 1) maintained by the oilseeds research program of the NARC, Islamabad, were used for characterization through morphological and molecular markers. Plant material comprises of 26 cytoplasmic sterility system (CMS) lines, 20 maintainer lines, 31 restorer lines, and 32 self-pollinated lines planted under open field conditions at the NARC, Islamabad, according to the conditions of the augmented block design.

### Data collection/phenotyping

Data on the morphological attributes including, plant height, stem curvature, number of leaves per plant, leaf area, time taken to flower initiation and flower completion, head diameter, seed yield per plot, and 100-seed weight were collected by the following standard procedures.

### DNA extraction and genotyping with SSR markers

For the molecular marker-based genotyping of 109 sunflower genotypes, gDNA was extracted from the leaves of a 12-day-old seedlings using the cetyl trimethyl ammonium bromide (CTAB) method of Saghai-Maroof et al. (1984). Extracted DNA was then diluted with 50 μl of TE buffer. DNA was run on 1% agarose gel to determine purity and concentration. DNA fingerprinting of the sunflower material was conducted through 40 SSR markers (Supplementary Table 2) selected from the sunflower linkage map (Saghai-Maroof et al., 1984). DNA fragments amplified by the respective SSR primers were designated as a unit trait with 1 for the presence and 0 for the absence, therefore, generating a binary matrix dataset.

### Diversity analysis

Genetic diversity parameters such as polymorphic information content (PIC) values were calculated using Power Marker program, number of effective alleles, Shannon information index, and pair-wise Nei's genetic distance among groups was determined through GeneAlEx 6.5 software. Furthermore, the binary matrix generated by the SSR genotyping, was then employed to construct a dendrogram cluster analysis using the Jaccard's similarity index for finding the heterotic groups in the studied sunflower germplasm based on the genetic distance among them.

### Statistical analysis

Collected data were analyzed using the univariate statistics, i.e., means, range, standard deviation, and coefficient of variation in MS Excel worksheet for observing the presence of genetic diversity that would warrant further statistical procedures. Multivariate statistics, i.e., principal component analysis (PCA) and cluster analysis were than conducted through R-Studio (v.1.3.134) to figure out the presence/absence of any kind of heterotic grouping pattern in the sunflower lines under examination.

## Results

The results of the univariate descriptive statistics of agro-morphological parameters, i.e., days to flower initiation, days to flower completion, plant height, stem curvature, number of leaves per plant, leaf area, seed yield per plant, and 100-seed weight. Minimum and maximum values of all the studied nine morphological traits of sunflower plants showed a considerable amount of diversity in the studied sunflower genotypes pool in open field conditions (Table 1). Range, mean, variance, standard deviation, and coefficient of variability (CV%) were calculated and an appreciable content of the genetic divergence was observed in the studied plant material.

**Table 1:** Range, mean, variance, standard deviation, and coefficient of variation (%) of nine morphological traits.

**Traits**	**Range**	**Mean**	**Variance**	**St. Deviation**	**C.V%**
Days to flower initiation	69–94	79.86	34.23	5.85	7.32
Days to flower completion	72–102	91.45	66.06	8.12	8.88
Plant height	121.8–246.25	185.46	736.2	27.13	14.63
Stem curvature	15.5–36.7	27.68	29.75	5.45	19.70
Head diameter	9.25–20.9	14.50	8.63	2.93	20.26
Leaves per plant	22–41.89	30.84	15.95	3.99	12.94
Leaf area	139.08–276.48	202.31	1,531.82	39.13	19.34
100-seed weight	2.76–6.94	4.92	0.97	0.98	20.08
Seed yield per plant	23.9–62.7	37.14	53.44	7.31	19.68

Highest value of variance (1,531.28) was observed for leaf area (LA) followed by PH (736.2), while for HSW lowest variance magnitude of 0.97 was recorded. Similarly, standard deviation of LA (39.13) and PH (27.13) was also the highest among all the nine morphological traits. The CV% of these two traits, i.e., LA and PH, was also above 20%, while the rest of the seven traits expressed a CV% of <20%.

### Cluster analysis

**Figure 1:**
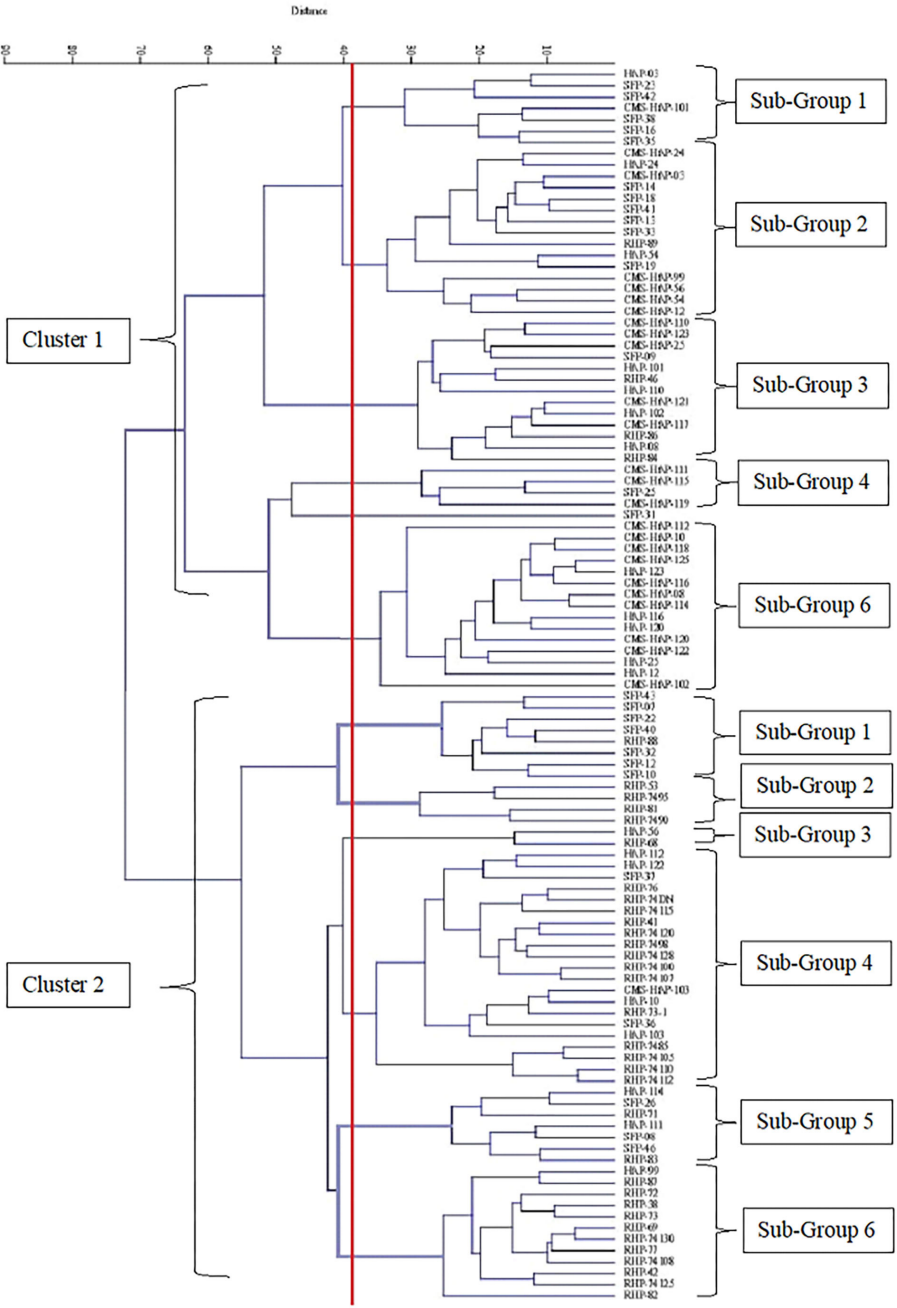
Cluster diagram constructed on nine morphological data of 109 sunflower genotypes.

Cluster diagram was generated from agro-morphological data of nine parameters to visualize the level of divergence among the 109 sunflower genotypes that were evaluated. Euclidean distance matrix generated through UPGMA option of analysis was employed to construct the tree diagram through the statistical software PAST version 3.14. Tree diagram showed two distinct clusters of sunflower genotypes (Figure 1). Cluster 1 contains 55 genotypes while cluster 2 contains 54 lines and these major clusters could be further divided into 6 sub-clusters. Mean values of various sub-groups and groups are identified based on the dendrogram and presented in Table 2. Characteristics exhibited by genotypes grouped in cluster 1 are early flowering, tall plant height, greater stem curvature, greater head diameter, higher number of leaves per plant, higher value for hundred seed weight, and greater seed yield per plant than cluster 2.

**Table 2:** Mean values of clusters and sub-groups for dendrogram constructed based on nine morphological traits.

**Parameters**	**Cluster 1**	**Sub-group 1**	**Sub-group-2**	**Sub-group-3**	**Sub-group-4**	**Sub-group-5**	**Sub-group-6**
DFI	74.673	73.714	75.133	75.385	72.250	69.000	75.067
DFC	84.218	85.143	86.667	85.385	78.000	77.000	82.467
PH	172.679	183.343	187.107	178.764	122.150	117.500	165.153
SC	30.585	30.310	31.468	31.104	29.713	15.500	30.618
HD	15.468	15.504	14.947	15.140	16.175	13.250	16.215
L/P	33.171	35.514	36.150	33.491	28.350	26.000	30.585
LA	204.975	190.441	209.298	223.310	203.373	251.240	188.886
HSW	5.361	5.752	5.407	5.361	5.192	6.340	5.114
SY/P	40.204	38.591	44.594	38.738	39.347	35.482	38.378
**Parameters**	**Cluster 2**	**Sub-group 1**	**Sub-group-2**	**Sub-group-3**	**Sub-group-4**	**Sub-group-5**	**Sub-group-6**
DFI	78.593	74.000	81.250	74.500	80.810	77.857	78.000
DFC	89.519	84.500	98.750	84.000	91.095	86.429	89.750
PH	169.173	179.156	151.800	162.000	177.852	167.171	155.483
SC	27.692	33.466	21.050	31.100	26.586	29.717	26.242
HD	12.582	15.219	10.700	15.150	12.091	15.079	10.425
L/P	32.216	33.813	28.613	29.700	34.867	28.786	30.135
LA	186.297	227.045	217.643	185.210	177.962	204.457	152.855
HSW	4.427	5.527	3.953	4.703	4.292	4.650	3.911
SY/P	33.373	37.222	29.252	38.562	32.535	38.097	30.027

DFI, Days to flower initiation; DFC, days to flower completion; PH, Plant height; SC, Stem curvature; HD, Head diameter; L/P, Number of leaves per plant; LA, Leaf area; HSW, Hundred seed weight; SY/P, Seed yield per plant.

Further grouping of cluster 1 into sub-clusters at 40% linkage distance showed that this cluster contained 6 sub-clusters (Table 2). The lowest mean value for days to flower initiation was observed for sub-group 5 (69) while genotypes with late initiation of flowering were grouped in sub-group 3 (75.385). Likewise, minimum value for days to 100% flowering was showed by sub-group 5 while sub-group 2 had the genotypes that took maximum number of days to complete flowering in cluster 1 (86.67). Sub-group 2 recorded the highest mean value for plant height while the genotypes with short stature were grouped in sub-group 4. Head diameter, a major yield influencing character in sunflower, was observed to be greater in sub-group 6 (16.215) and lowest in sub-group 5 (13.25). Genotypes with more number of leaves were grouped in sub-group 2 (36.15) and sub-group 1 (35.51), respectively, while those with less number of leaves per plant were clustered in sub-group 5 (26) and sub-group 4 (28.35). Sub-group 5 (6.34) and sub-group 1 (5.75) recorded the highest mean value of a hundred seed weight in cluster 1. Seed yield per plant was the highest among the genotypes of sub-group 2 (44.59) and lowest for sub-group 5 (35.48).

In total, six sub-groups in cluster 2 were noted at 40% linkage distance. Days to flower initiation was found minimum for sub-group 1 (74) and maximum for sub-group 2 (81.25). Sub-group 1 and sub-group 4 showed the maximum cluster mean value for plant height (179.516 and 177.85) and sub-group 2 (151.8) and sub-group 6 (155.48) with the minimum mean value. The highest cluster mean value for stem curvature was exhibited by sub-group 1 (33.466) and lowest value (21.05) by sub-group 2. The highest cluster means for head diameter 15.21 and 15.15 was noted in sub-groups 1 and 3, respectively, whereas, minimum value of 10.7 and 10.42 was observed for sub-groups 2 and 6, respectively. Sub-group 4 had shown the highest cluster mean for number of leaves per plant (34.867) and sub-group 2 (28.613) and sub-group 5 (28.786) with the lowest. Mean value for average leaf area was found to be the lowest in sub-group 6 (152.85) and highest in sub-group 1 (227.045). Maximum mean value for hundred seed weight (5.527) was observed in sub-group 1 among seven sub-groups, whereas sub-group 6 (3.91) and sub-group 2 (3.95) showed the lowest mean value for hundred seed weight. Sub-group 3 possessed the highest seed yield per plant mean (38.562) followed by sub-group 5 (38.097) while sub-group 2 had the lowest mean value for seed yield per plant (29.252).

### Principal component analysis

**Figure 2:**
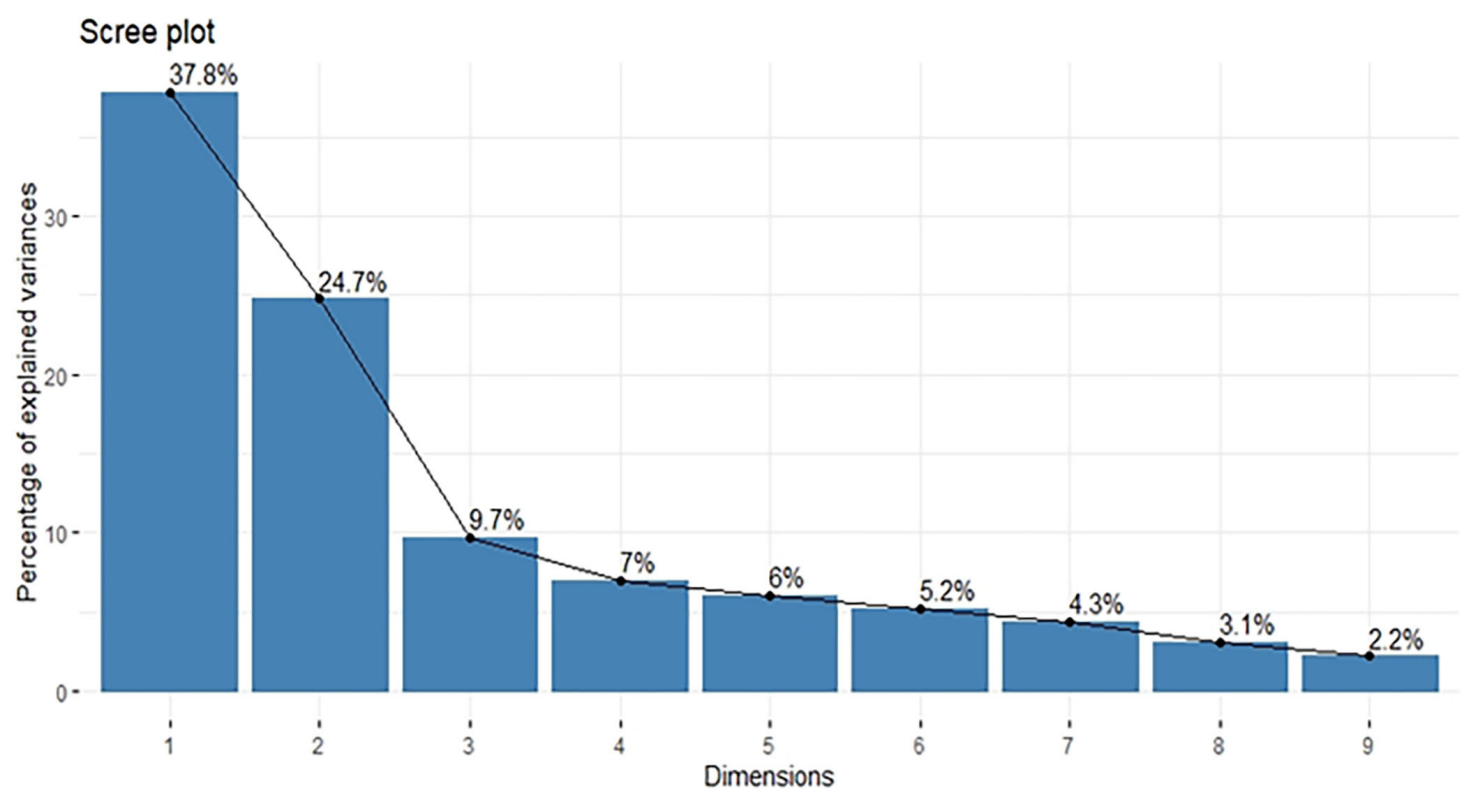
Scree plot diagram of nine principal components with percentage of variance explained by each principal component.

Principal component analysis was performed using standardized data of agro-morphological traits collected from 109 sunflowers. Scree plot diagram (Figure 2) showed the Eigen values of agro-morphological traits studied for the 109 lines. The Eigen values showed that first two components had Eigen values >1. First eigenvector was responsible for 37.78% of all the variability observed among all the sunflower lines. Factor loading tables (Table 3) results showed that except days to flower initiation and days to flower completion rest of all the traits had a positive contribution toward the first principal component. Major traits that contributed toward the component include head diameter, hundred seed weight, and seed yield per plant. The second principal component contributed 24.748% toward the total variability. Factor loading table results depicted that only head diameter had a negative contribution toward the second PC. Major weight contributing traits with the second principal component include the number of leaves per plant, plant height, and days to flower completion.

**Table 3:** Factor loadings of various morphological traits toward nine principal components of 109 sunflower genotypes.

**Traits**	**PC-1**	**PC-2**	**PC-3**	**PC-4**	**PC-5**	**PC-6**	**PC-7**	**PC-8**	**PC-9**
DFI	−0.051	0.045	0.468	0.284	0.046	−0.010	0.830	−0.072	0.019
DFC	−0.049	0.097	0.704	0.340	0.269	−0.090	−0.525	0.147	0.000
PH	0.398	0.911	−0.048	−0.048	−0.046	−0.066	0.020	−0.010	0.001
SC	0.028	−0.007	−0.311	0.073	0.876	−0.317	0.088	−0.146	−0.024
HD	0.030	−0.004	−0.181	0.135	0.069	0.011	0.140	0.951	−0.140
L/P	0.027	0.074	−0.058	0.114	0.300	0.939	−0.019	−0.064	−0.041
L/A	0.910	−0.390	0.132	−0.025	0.018	0.010	0.011	−0.005	−0.004
HSW	0.011	−0.001	−0.045	0.023	0.042	0.033	0.005	0.129	0.989
SY/P	0.073	−0.022	−0.362	0.874	−0.246	−0.064	−0.091	−0.164	−0.003

DFI, Days to flower initiation; DFC, days to flower completion; PH, Plant height; SC, Stem curvature; HD, Head diameter; L/P, Number of leaves per plant; LA, Leaf area; HSW, Hundred seed weight; SY/P, Seed yield per plant.

**Figure 3:**
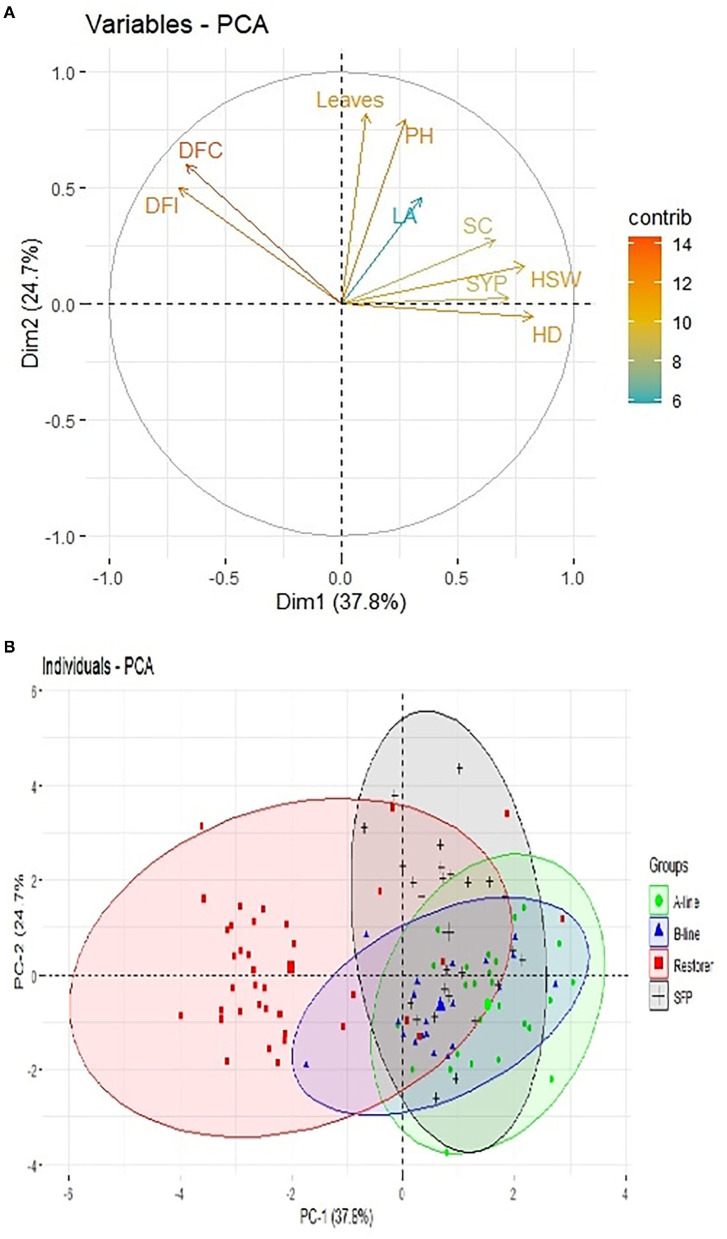
Scatter plot diagram of nine morphological traits and 109 sunflower genotypes. **(A)** Variable bi-plot showing contribution of nine traits under study in variability analysis. **(B)** 109 sunflower genotypes grouping pattern in principal component analysis.

Principal component analysis scatter plot diagram (Figure 3) further illustrated the diversity among the sunflower material studied based on the first two principal components. Based on the nine agro-morphological traits, the genotypes were scattered over four quadrants. Genotypes that cluster close to each other share some common features while those having diversity among each other scatter away from the bi-plot. It is evident from the bi-plot diagram that most of the restorer lines grouped close to each other and separately from rest of the sunflower lines, while CMS, B-lines and self-pollinated lines clustered together suggesting a close relationship among themselves.

### Genetic diversity analysis at molecular level

**Figure 4:**
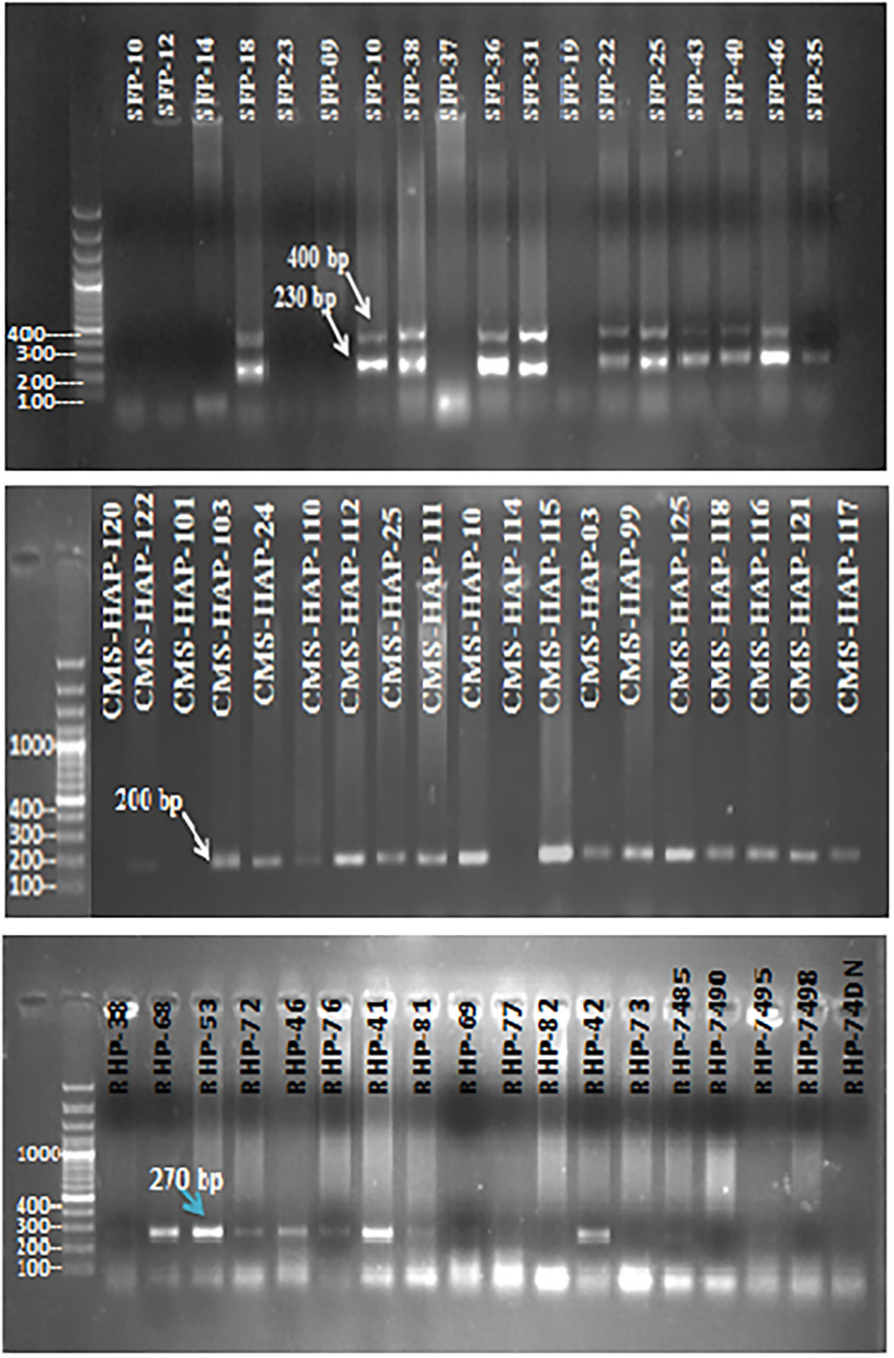
Gel electropherogram of SSR marker (ORS-1024), (ORS-1085), and (ORS-613) for some sunflower genotypes.

For genotypic/molecular characterization of 109 sunflower lines 40 SSR markers were employed. Selected markers were spread throughout the sunflower genome. Sunflower genetic map constructed by Tang et al. (2002) that 459 SSR markers showed 17 clusters, corresponding to 17 haploid chromosome numbers of Helianthus annuus. Markers, for the present study, amplified a total of 84 polymorphic bands (Figure 4). Each amplified DNA band was treated as a unit characteristic of sunflower studied panel, with 0 for absence and 1 for presence of a particular band in representative genotypes. Binary matrix of SSR profiling was used for cluster analysis to gain an insight divergence among the sunflower material at the molecular level through the Jaccard's similarity index by UPGMA.

In total, 40 SSR primers employed in this study to investigate the genetic diversity among 109 sunflower accessions amplified a total of 84 scorable bands. The lowest and the highest PIC values observed was 0.018–0.488 for ORS-1088 and ORS-366, respectively, expected heterozygosity ranged from 0.019 to 0.0468 as showed by the primer pairs ORS-599 and ORS-700 and ORS-605. Number of alleles produced by these 40 SSR primer pairs was from 1 to 4, with effective allele number ranging from 2.882 (ORS-605 and ORS-700) to 1.020 (ORS-845, ORS-511, and ORS-599), while Shannon Information index of the utilized SSR primer pairs showed the highest value of 0.661 (ORS-700 and ORS-605) and the lowest value of 0.041 (ORS-599, ORS-511, and ORS-845). The results of the genetic diversity estimates suggested the presence of a moderate genetic diversity among the studied pool of sunflower genotypes (Table 4). Pair wise genetic distance estimates according to the Nei's gene distance index (Table 4) showed that CMS-lines and SFP (self-pollinated lines) had the highest variation between them (0.093), while restore lines and SFP exhibited the lowest diversity among them (0.019).

**Table 4:** Genetic diversity estimates as revealed by 40 SSR primers and Pairwise Nei's Genetic distance among four sunflower populations.

**SSR primers**	**Ne**	**I**	**PIC**	**Na**	**He**
ORS-811	1.492	0.464	0.434	4.000	0.301
ORS-366	1.542	0.514	0.488	2.000	0.336
ORS-769	1.513	0.487	0.375	2.000	0.316
ORS-1085	1.879	0.660	0.354	1.000	0.467
ORS-307	1.841	0.642	0.371	2.000	0.451
ORS-1024	1.866	0.656	0.375	2.000	0.463
ORS-613	1.784	0.620	0.368	2.000	0.431
ORS-453	1.848	0.639	0.454	1.000	0.449
ORS-1068	1.466	0.433	0.447	3.000	0.278
ORS-1088	1.133	0.133	0.018	1.000	0.087
ORS-605	2.882	0.661	0.300	1.000	0.468
ORS-543	1.669	0.581	0.167	1.000	0.394
ORS-371	1.133	0.133	0.018	1.000	0.087
ORS-1053	1.136	0.215	0.224	1.000	0.115
ORS-882	1.377	0.303	0.360	2.000	0.209
ORS-617	1.108	0.198	0.167	1.000	0.096
ORS-857	1.087	0.138	0.136	1.000	0.073
ORS-752	1.075	0.137	0.136	1.000	0.066
ORS-924	1.035	0.060	0.094	2.000	0.031
ORS-337	1.167	0.148	0.036	1.000	0.100
ORS-533	1.243	0.171	0.088	1.000	0.123
ORS-608	1.632	0.567	0.422	1.000	0.381
ORS-1256	1.136	0.215	0.224	1.000	0.115
ORS-400	1.133	0.133	0.018	1.000	0.087
ORS-700	2.882	0.661	0.300	1.000	0.468
ORS-830	1.256	0.241	0.072	2.000	0.157
ORS-599	1.020	0.041	0.036	1.000	0.019
ORS-795	1.108	0.198	0.167	1.000	0.096
ORS-433	1.451	0.398	0.411	3.000	0.262
ORS-697	1.058	0.084	0.251	1.000	0.047
ORS-1040	1.676	0.567	0.337	2.000	0.384
ORS-879	1.083	0.104	0.288	1.000	0.062
ORS-781	1.108	0.198	0.167	1.000	0.096
ORS-511	1.020	0.041	0.036	1.000	0.019
ORS-1086	1.619	0.556	0.152	1.000	0.372
ORS-562	1.256	0.241	0.072	1.000	0.157
ORS-768	1.406	0.317	0.255	1.000	0.222
ORS-1064	1.155	0.175	0.460	1.000	0.106
ORS-845	1.020	0.041	0.036	1.000	0.019
ORS-495	1.108	0.198	0.167	1.000	0.096
	CMS-lines	Maintainer-lines	Restorer-lines	SFP-lines	
**Pairwise Nei's Genetic distance among four sunflower populations**					
CMS-lines	0.000				
Maintainer-lines	0.088	0.000			
Restorer-lines	0.084	0.060	0.000		
SFP-lines	0.093	0.085	0.019	0.000	

PIC, Polymorphic information content; He, Expected heterozygosity; Na, number of alleles; Ne, Number of effective alleles; I, Shannon Information Index; SFP, Self-pollinated lines.

**Figure 5:**
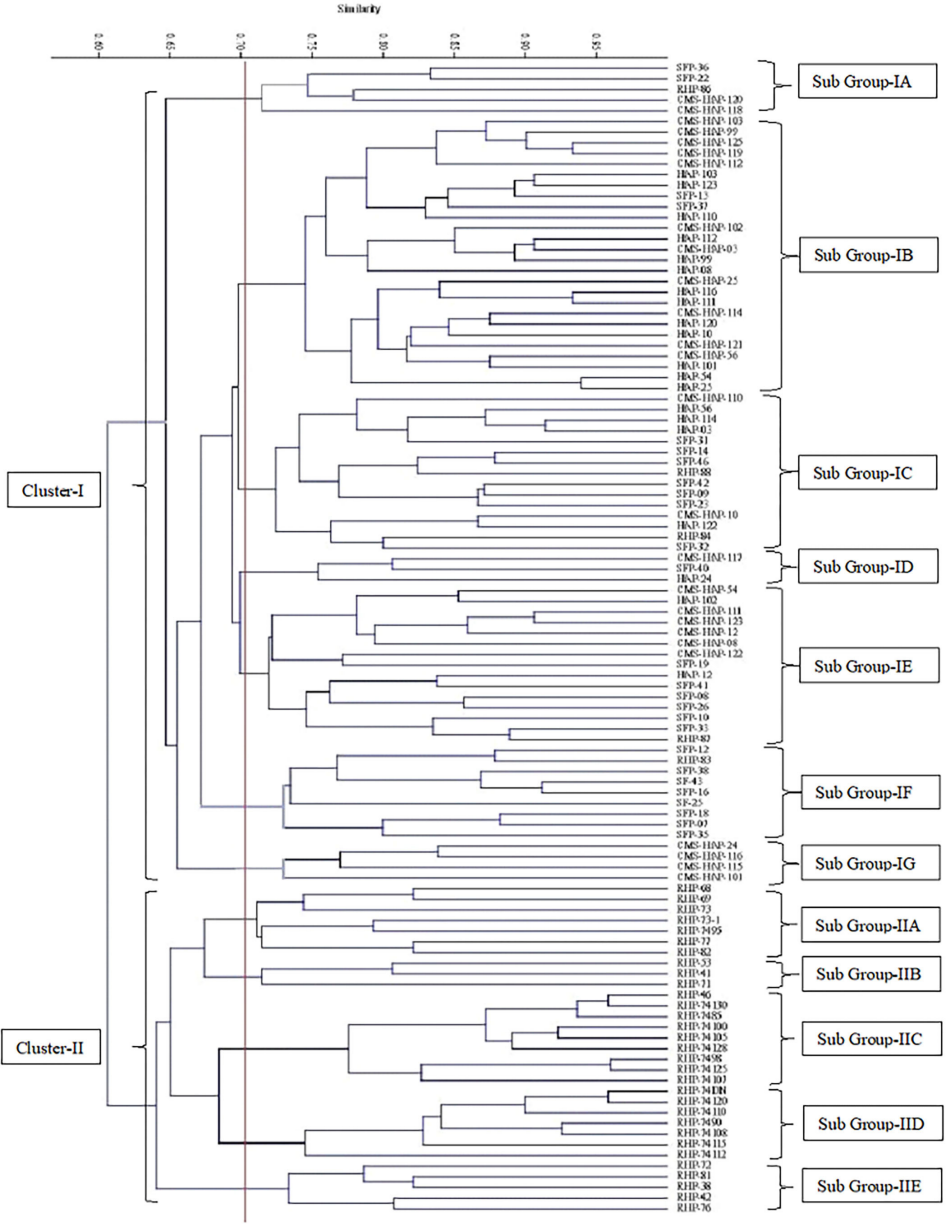
Cluster diagram constructed on 40 SSR molecular markers data of 109 sunflower genotypes.

The tree diagram (Figure 5) generated, splits the lines into two major clusters, where one contains only restorer and the other group contains all CMS, maintainer, SFP, and 7 restorer lines as well. The cluster 1 contains 31 genotypes in all and can be sub-divided into five smaller groups. Group 1A had 9 genotypes, while the groups 1B, 1C, 1D, and 1E contains 7, 9, 3, and 5 genotypes, respectively (Table 5). The other major cluster can also be sub-divided into 7 smaller groups, wherein, sub-group 2A contains 15 genotypes followed by sub-group as 2B, 2C, 2D, 2E, 2F, and 2G in which 11, 15, 19, 9, 5, and 4 sunflower accessions grouped together, respectively.

**Table 5:** Grouping of 109 sunflower genotypes based on SSR markers dendrogram.

**Cluster**	**Sub-group**	**Genotypes**	**No. of genotypes**	**Percent %**
I	A	SFP-36, SFP-22, RHP-86, CMS-HAP-120, CMS-HAP-118	5	4.59
	B	CMS-HAP-103, CMS-HAP-99, CMS-HAP-125, CMS-HAP-119, CMS-HAP-112, HAP-103, HAP-123, SFP-13, SFP-37, HAP-110, CMS-HAP-102, HAP-112, CMS-HAP-03, HAP-99, HAP-08, CMS-HAP-25, HAP-116, HAP-111, CMS-HAP-114, HAP-120, HAP-10, CMS-HAP-121, CMS-HAP-56, HAP-101, HAP-54, HAP-25	27	24.77
	C	CMS-HAP-110, HAP-56, HAP-114, HAP-03, SFP-31, SFP-14, SFP-46, RHP-88, SFP-42, SFP-09, SFP-23, CMS-HAP-10, HAP-122, RHP-84, SFP32	15	13.76
	D	CMS-HAP-117, SFP-40, HAP24	3	2.75
	E	CMS-HAP-54, HAP-102, CMS-HAP-111, CMS-HAP-123, CMS-HAP-12, CMS-HAP-08, CMS-HAP-122, SFP-19, HAP-12, SF-41, SFP-08, SFP-26, SFP-10, SFP-33, RHP-87	15	13.76
	F	SFP-12, RHP-83, SFP-38, SFP-43, SFP-16, SFP-25, SFP-18, SFP-07, SFP-35	9	8.26
	G	CMS-HAP-24, CMS-HAP-116, CMS-HAP-115, CMS-HAP-101	4	3.67
II	A	RHP-82, RHP-77, RHP-7495, RHP-73-1, RHP-73, RHP-69, RHP-68	7	6.42
	B	RHP-71, RHP-53, RHP-41	3	2.75
	C	RHP-46, RHP-74130, RHP-7485, RHP-74100, RHP-74105, RHP-74128, RHP-7498, RHP-74125, RHP-74107	9	8.26
	D	RHP-74120, RHP-74115, RHP-74108, RHP-74112, RHP-74DN, RHP-7490, RHP-74110	7	6.42
	E	RHP-76, RHP-81, RHP-72, RHP-42, RHP-38	5	4.59

## Discussion

Germplasm collections are valuable resources for crop improvement. However, to fully unlock their potential, it is critical to have detailed knowledge about the amount and the distribution of the genetic diversity available within collections (Tang et al., 2002). Genetic diversity among sunflower is based on the genetic distance (GD) among them (Filippi et al., 2020). Previous scientific studies attempted to figure out the relationship between the performance of F_1_ and GD among parents revealed a significant association between heterosis and GD of parents in different crops including wheat (Geng et al., 2021), rapeseed (Nie et al., 2019; Ahmad et al., 2020; Aris et al., 2020), cacao (Nikzad et al., 2020), maize (Mustiga et al., 2018), pearl millet (Nyaga et al., 2020), and cotton (Filippi et al., 2020).

Genotypes represented a significant amount of variation for flowering traits (initiation and completions) with early flowering genotypes would be more advantageous in their usage for breeding high-yielding sunflower hybrids as they tend to have more time window for grain filling period thus leading to higher seed weight (Gupta et al., 2018) biomass-related traits including plant height, stem curvature, number of leaves per plant, and leaf area exhibit quantitative inheritance and plants with moderate stem height and head drooping (stem curvature) up to 35% are high-yielding owing to their ability to withstand lodging, early maturity characteristic and less bird damage (Azad et al., 2016). In the previous sunflower morphological diversity profiling, similar type of high-degree variations have been reported, validating the findings of the present study (Khan, 2006; Arshad et al., 2007; Gupta et al., 2018).

Short stature plant lines could be used in further sunflower improvement programs as tall hybrids mature late than short stature hybrids (Rani, 2016; Ram et al., 2018; Farooq et al., 2021). More leaves per plant and more leaf area could be good indicator of high-yield potential sunflower genotypes as these traits provide more surface area for the photosynthetic activity, thus, leading to accumulation of more photosynthates and more yield per plant (Enns, 1959). Environmental effects contribute more toward head diameter. Head size is found to be highly influenced by plant population, moisture, and nutrient contents of the soil. Usually, a large sunflower head was found to have a wider sterile zone area thus a medium-size head is desirable in sunflower hybrid breeding (Azad et al., 2016; Ibrar et al., 2018; Amiteye et al., 2021). Appreciable genetic variability among the genotypes studied was found for seed yield per plant which shows that it can be utilized in further breeding programs.

Analyzing genetic diversity through multivariate statistics involved the use of cluster analysis, and principal component analysis for grouping genotypes based on the similarities and differences among them (Peeters and Martinelli, 1989; Nasreen et al., 2011). According to Mohammadi and Prasanna (2003), many researchers have previously successfully utilized these multivariate techniques for diversity analysis in different crops such as barley (Ruzdik et al., 2015), wheat (Cross, 1992), sorghum (Hailu et al., 2006), and sunflower (Ayana and Bekele, 1999). Results of cluster analysis grouped CMS and restorer lines separately. This may be because both types of sunflowers had a different center of origin and breeding histories. Distinct clustering pattern in sunflower, wherein CMS and restorer lines clustered separated from each other illustrating a different evolutionary pathway for each type (Lochner, 2011; Avin et al., 2013). Clustering of genotypes into different groups was based on their specific characteristics (Table 2) (Kholghi et al., 2011; Shamshad et al., 2014; Golabadi et al., 2015). Genotypes that were early in the flowering were taller, larger in head size, high hundred seed weight, and produced more seed yield than those that were late in flowering thus having less time for seed development and filling, consequently leading to low seed yield. Similarly, previous findings are also in accordance with the present study wherein clusters showing higher mean values for seed yield also exhibited higher mean values for head diameter and 100-seed weight (Kumar et al., 2008; Punitha et al., 2010).

Another multivariate statistic used in the present study is PCA, which allows the clustering or grouping of genotypes and also transforms the large set of variables into the smaller non-correlated set. PCA is being widely utilized for summarizing a larger set of parameters, thereafter, measuring their importance on each axis or component by its weight, which is associated with the axis (Chandirakala and Manivannan, 2014). Biplot analysis of the PCA was first developed by Gabriel (Inacio et al., 2002), and it can interpret multiple variables in the function of different treatments in the same graphic (Gabriel, 1971). Results of the present experiments revealed that while selecting the parents the characters that contributed positively to the first two principal components should be considered. As the first principal component contributed 37.78% and the maximum factor loading observed for the first principal components were found to be head diameter, hundred seed weight, and seed yield per plant. Hence, during the selection of parents to be used in sunflower hybrid breeding programs, traits contributed positively to factor loadings values should be prioritized (Arshad et al., 2010; Akinwale et al., 2014; Mustafa et al., 2015). The results of the present investigation are in accordance with the previous findings of Masvodza et al. (2015), who also reported that cms and restorer lines grouped in different planes when plotted on a 2D graph.

PCA can be used efficiently in cultivar development programs as it showed a significant amount of correspondence with the conventional breeding methods (Tabrizi et al., 2009). The principal component analysis is a powerful method of data dimensionality reduction thus removing the correlation among the components (Ghafari, 2004). Similarly, it has been reported that PCA is an effective technique that can be used for the selection of promising genotypes with desirable characters among identified clusters (Arshad et al., 2006; Muhammad et al., 2009).

Markers exhibiting high PIC values such as ORS-605 and ORS-700 are generally considered suitable for analyzing the genetic diversity among germplasms. In sunflower diversity analysis conducted by paper ssr1, wherein two SSR primer pairs (Ha806-ar and ORS-31) were identified as suitable for diversity profiling among sunflower germplasm, likewise, Sahranavard et al. (2015) also observed markers HA3040 and ORS-733 that showed high PIC values are appropriate for analyzing genetic variability diversity in oily sunflower. Results of diversity profiling through SSR markers although showed a moderate level of genotypic diversity, however, these findings of PIC values are in close concurrence to those obtained by Lochner (2011) (0.06–0.75), Erasmus (2008) (0.17–0.80), and Darvishzadeh et al. (2010) (0.09–0.62). Number of alleles in this present study was 1–4 with an average number of alleles was 2.1. Antonova et al. (2006) used 10 SSR primer pairs in the genetic diversity of 17 sunflower inbred lines and hybrids and recorded an average of 2.2 alleles.

Molecular characterization through SSR also splits the sunflower lines into two major groups of Restore and CMS lines as in the case of morphological characterization. Various previous researchers who used SSR markers for sunflower characterization at molecular data also found that sunflower lines had a clear tendency of grouping restorer and CMS lines into different clusters. For examining the genetic diversity in sunflower SSR, markers are a powerful as it reveals high level of polymorphism among sunflower lines (Lochner, 2011; Zia et al., 2014; Suresha et al., 2017; Ijaz et al., 2020).

## Conclusion

The results of the genetic diversity and grouping/clustering based on the UPGMA analysis of molecular and morphological markers data depicted a significant amount of genetic variability that exists among the studied sunflower genetic resources. Both dendrograms revealed a clear and distinct pattern of the heterotic grouping in the crop. This heterotic grouping is based on the genetic distance among the studied material and genetic distance has been found useful in the selection of parental lines for hybrid breeding programs in various crops such as wheat, cotton, pearl millet, etc. It is inferred that mating between members of different heterotic groups could yield better and out-performing hybrids. Therefore, as every member of heterotic groups is representative of the whole group, one genotype with the highest seed yield per plant should be selected from each identified heterotic group and utilized in the hybrid breeding program through L × T mating design to study the efficiency of genetic distance and seed yield per plant being a predictor of hybrids performance in cultivated sunflower. Genotypes that could be potentially exploited from these identified heterotic groups include, CMS-HAP-12, CMS-HAP-54, CMS-HAP-56, CMS-HAP-99, CMS-HAP-111, and CMS-HAP-112 as female parents and RHP-38, RHP-41, RHP-53, RHP-68, RHP-69, and RHP-71 as a restorer or male parents for hybrid development.

## Data availability statement

The original contributions presented in the study are included in the article/Supplementary material, further inquiries can be directed to the corresponding author/s.

## Author contributions

All authors listed have made a substantial, direct, and intellectual contribution to the work and approved it for publication.

## Funding

This research was funded by the Higher Education Commission (HEC) of Pakistan through Indigenous Ph.D. Fellowship to DI (HEC Fellowship No. 112-32932-2Av1-083).

## Conflict of interest

The authors declare that the research was conducted in the absence of any commercial or financial relationships that could be construed as a potential conflict of interest.

## Publisher's note

All claims expressed in this article are solely those of the authors and do not necessarily represent those of their affiliated organizations, or those of the publisher, the editors and the reviewers. Any product that may be evaluated in this article, or claim that may be made by its manufacturer, is not guaranteed or endorsed by the publisher.
