# Supplementary material for: Molecular markers-based DNA fingerprinting coupled with morphological diversity analysis for prediction of heterotic grouping in sunflower (Helianthus annuus L.)
Source: Front Plant Sci. 2022 Jul 28;13:916845. doi: 10.3389/fpls.2022.916845 (PMC9366523; doi:10.3389/fpls.2022.916845)
Supplement: Supplementary file 1 [file Data_Sheet_1.docx]

**Table S1.** List of Sunflower Accessions used in present study

| S. No. | Accession No. | Source | S. No. | Accession No. | Source |
| --- | --- | --- | --- | --- | --- |
| 1 | CMS-HAP-12 | *NARC, Islamabad* | 56 | RHP-38 | *NARC, Islamabad* |
| 2 | CMS-HAP-56 | *NARC, Islamabad* | 57 | RHP-77 | *NARC, Islamabad* |
| 3 | CMS-HAP-101 | *NARC, Islamabad* | 58 | RHP-82 | *NARC, Islamabad* |
| 4 | CMS-HAP-54 | *NARC, Islamabad* | 59 | RHP-42 | *NARC, Islamabad* |
| 5 | CMS-HAP-103 | *NARC, Islamabad* | 60 | RHP-73 | *NARC, Islamabad* |
| 6 | CMS-HAP-24 | *NARC, Islamabad* | 61 | RHP-74DN | *NARC, Islamabad* |
| 7 | CMS-HAP-110 | *NARC, Islamabad* | 62 | RHP-7485 | *NARC, Islamabad* |
| 8 | CMS-HAP-112 | *NARC, Islamabad* | 63 | RHP-7490 | *NARC, Islamabad* |
| 9 | CMS-HAP-25 | *NARC, Islamabad* | 64 | RHP-7495 | *NARC, Islamabad* |
| 10 | CMS-HAP-111 | *NARC, Islamabad* | 65 | RHP-7498 | *NARC, Islamabad* |
| 11 | CMS-HAP-10 | *NARC, Islamabad* | 66 | RHP-74100 | *NARC, Islamabad* |
| 12 | CMS-HAP-114 | *NARC, Islamabad* | 67 | RHP-74105 | *NARC, Islamabad* |
| 13 | CMS-HAP-115 | *NARC, Islamabad* | 68 | RHP-74107 | *NARC, Islamabad* |
| 14 | CMS-HAP-03 | *NARC, Islamabad* | 69 | RHP-74108 | *NARC, Islamabad* |
| 15 | CMS-HAP-99 | *NARC, Islamabad* | 70 | RHP-74110 | *NARC, Islamabad* |
| 16 | CMS-HAP-125 | *NARC, Islamabad* | 71 | RHP-74112 | *NARC, Islamabad* |
| 17 | CMS-HAP-118 | *NARC, Islamabad* | 72 | RHP-74115 | *NARC, Islamabad* |
| 18 | CMS-HAP-116 | *NARC, Islamabad* | 73 | RHP-74120 | *NARC, Islamabad* |
| 19 | CMS-HAP-121 | *NARC, Islamabad* | 74 | RHP-74125 | *NARC, Islamabad* |
| 20 | CMS-HAP-117 | *NARC, Islamabad* | 75 | RHP-74128 | *NARC, Islamabad* |
| 21 | CMS-HAP-122 | *NARC, Islamabad* | 76 | RHP-74130 | *NARC, Islamabad* |
| 22 | CMS-HAP-120 | *NARC, Islamabad* | 77 | RHP-71 | *NARC, Islamabad* |
| 23 | CMS-HAP-123 | *NARC, Islamabad* | 78 | SFP-14 | *NARC, Islamabad* |
| 24 | CMS-HAP-102 | *NARC, Islamabad* | 79 | SFP-12 | *NARC, Islamabad* |
| 25 | CMS-HAP-08 | *NARC, Islamabad* | 80 | SFP-10 | *NARC, Islamabad* |
| 26 | CMS-HAP-119 | *NARC, Islamabad* | 81 | SFP-40 | *NARC, Islamabad* |
| 27 | HAP-12 | *NARC, Islamabad* | 82 | SFP-42 | *NARC, Islamabad* |
| 28 | HAP-56 | *NARC, Islamabad* | 83 | SFP-38 | *NARC, Islamabad* |
| 29 | HAP-101 | *NARC, Islamabad* | 84 | SFP-18 | *NARC, Islamabad* |
| 30 | HAP-54 | *NARC, Islamabad* | 85 | SFP-36 | *NARC, Islamabad* |
| 31 | HAP-103 | *NARC, Islamabad* | 86 | SFP-31 | *NARC, Islamabad* |
| 32 | HAP-24 | *NARC, Islamabad* | 87 | SFP-37 | *NARC, Islamabad* |
| 33 | HAP-110 | *NARC, Islamabad* | 88 | SFP-24 | *NARC, Islamabad* |
| 34 | HAP-112 | *NARC, Islamabad* | 89 | SFP-09 | *NARC, Islamabad* |
| 35 | HAP-25 | *NARC, Islamabad* | 90 | SFP-41 | *NARC, Islamabad* |
| 36 | HAP-102 | *NARC, Islamabad* | 91 | SFP-19 | *NARC, Islamabad* |
| 37 | HAP-10 | *NARC, Islamabad* | 92 | SFP-22 | *NARC, Islamabad* |
| 38 | HAP-114 | *NARC, Islamabad* | 93 | SFP-25 | *NARC, Islamabad* |
| 39 | HAP-116 | *NARC, Islamabad* | 94 | SFP-43 | *NARC, Islamabad* |
| 40 | HAP-123 | *NARC, Islamabad* | 95 | SFP-33 | *NARC, Islamabad* |
| 41 | HAP-111 | *NARC, Islamabad* | 96 | SFP-46 | *NARC, Islamabad* |
| 42 | HAP-99 | *NARC, Islamabad* | 97 | SFP-08 | *NARC, Islamabad* |
| 43 | HAP-122 | *NARC, Islamabad* | 98 | SFP-07 | *NARC, Islamabad* |
| 44 | HAP-120 | *NARC, Islamabad* | 99 | SFP-16 | *NARC, Islamabad* |
| 45 | HAP-03 | *NARC, Islamabad* | 100 | SFP-26 | *NARC, Islamabad* |
| 46 | HAP-08 | *NARC, Islamabad* | 101 | SFP-13 | *NARC, Islamabad* |
| 47 | RHP-68 | *NARC, Islamabad* | 102 | SFP-35 | *NARC, Islamabad* |
| 48 | RHP-72 | *NARC, Islamabad* | 103 | SFP-20 | *NARC, Islamabad* |
| 49 | RHP-53 | *NARC, Islamabad* | 104 | SFP-32 | *NARC, Islamabad* |
| 50 | RHP-73-1 | *NARC, Islamabad* | 105 | RHP-83 | *NARC, Islamabad* |
| 51 | RHP-46 | *NARC, Islamabad* | 106 | RHP-84 | *NARC, Islamabad* |
| 52 | RHP-76 | *NARC, Islamabad* | 107 | RHP-88 | *NARC, Islamabad* |
| 53 | RHP-41 | *NARC, Islamabad* | 108 | RHP-86 | *NARC, Islamabad* |
| 54 | RHP-81 | *NARC, Islamabad* | 109 | RHP-89 | *NARC, Islamabad* |
| 55 | RHP-69 | *NARC, Islamabad* |  |  |  |

**Table S2.** List of SSR markers used to study the association mapping and population structure in sunflower

| Primer Name | Linkage Group | Forward Sequence | Reverse Sequence |
| --- | --- | --- | --- |
| ORS-605 | 1 | CGCGTGATGTGACGATTATT | ACGGAGCAAAGTTTCGAGGT |
| ORS-543 | 1 | CCAAGTTTCAGTTACAATCCATGA | GGTCATTAGGAGTTTGGGATCA |
| ORS-371 | 1 | CACACCACCAAACATCAACC | GGTGCCTTCTCTTCCTTGTG |
| ORS-453 | 2 | CCTGTGAGCTACAATACTCCCACA | GATTCTGATTAGGCGGTGGT |
| ORS-1053 | 2 | TTTCATCACATTAGACCATAGACCA | GGCTTTCCTTCGTGGTTTGTAT |
| ORS-752 | 3 | CACTGATGAACAAGTGCGAGA | ATGATTCCCATACCCACCAA |
| ORS-924 | 3 | TAAATCGCCATACCACTCCATC | TATCAGCAGGAAGAACGCCTAAT |
| ORS-366 | 4 | AACCAACTGAGCATTCTTGTGA | GCGCTAGGTTAAAGAGGACAAA |
| ORS-1068 | 4 | AATTTGTCGACGGTGACGATAG | TTTTGTCATTTCATTACCCAAGG |
| ORS-337 | 4 | TTGGTTCATTCATCCTTGGTC | GGGTTGGTGGTTAATTCGTC |
| ORS-1024 | 5 | GGGAAGTGGGCTTGTCTATGTAT | AACACACCGAAATCACCTATGAA |
| ORS-533 | 5 | TGGTGGAGGTCACTATTGGA | AGGAAAGAAGGAAGCCGAGA |
| ORS-608 | 6 | CATGGAAAGCCGAGTTCTCT | CGTGCGTGATTAACATACCC |
| ORS-1256 | 6 | GATGTTGATGTTGGTGAAGTTGC | CTCCGTCACCTTAAGCACTTGTA |
| ORS-400 | 7 | CGAACCCGTCTGTACCGTTT | ACTTCGTTCACAAGGCACAA |
| ORS-700 | 7 | GTACCCACCACGCTTAACCA | AGTCTTCCACAGCAACGTCA |
| ORS-830 | 8 | CAAGTGCATTAGGTGGTTCTAACA | GCCCTCTGACTGTTGTATGACTG |
| ORS-599 | 8 | TTCCCTATCACACGCCTCTC | GAAAGGAAGTAGCGGTGGTG |
| ORS-882 | 9 | AAACCGGCATGTAAGATATTCG | ATCGGGAGCAGAAGAAGAGTATG |
| ORS-617 | 9 | GGTACTTGGTATTCATGGGTCAT | GACACCGCCAACTTAACACTT |
| ORS-795 | 9 | CGCTAGTTACACCGCAGATG | TGTCCACAGGTTGAAGATCG |
| ORS-613 | 10 | GTAAACCCTAGGTCAATTTGCAG | ATCTCCGGAAAACATTCTCG |
| ORS-1088 | 10 | ACTATCGAACCTCCCTCCAAAC | GGATTTCTTTCATCTTTGTGGTG |
| ORS-433 | 10 | CCGAGGTTTGATCGCTATTT | AGCGTTTGTGATTTGATTACGA |
| ORS-769 | 11 | GTTTATTTATGTAGAAATGTTCTGGAA | ATGTGGTGGTAAGGGTTGTTG |
| ORS-697 | 11 | TTGGGCTGTGGTTCCTTAAC | AAGAGATGGGAGTGTTGATGC |
| ORS-1085 | 12 | GACCTCAAGGCATGCTAACACTC | ACTAAGTGTGTGGACGGGGAAA |
| ORS-1040 | 12 | CTGCTGATCGTTTCTTGGATAGA | TGCTAATCCTTCTAATCAACTTCCAC |
| ORS-879 | 13 | GAACCTCCCTTTGTCTGCATATC | CTCCGGTTGCTGTTGATGTCT |
| ORS-781 | 13 | GTCAACCCATGACCCAAACC | GATGTGGAGGAGAGAGGGTGT |
| ORS-511 | 13 | TGGCTCAGATTAAGTTCACACAG | CGGGTTGCGAGTAACAGGTA |
| ORS-307 | 14 | CAGTTCCCTGAAACCAATTCA | GCAGTAGAAGATGACGGGATG |
| ORS-1086 | 14 | TTGTTTGTCGCACACTCAAGATT | ATTATCGGCACATCTTTGGATTT |
| ORS-857 | 15 | ACATCCGAACGAAGGACAATC | CAAGAAAGTATGTCACCCAATAGCA |
| ORS-562 | 15 | CACACACACAAACCCTAGCTCT | CAATCATATCGAGCACACATCA |
| ORS-768 | 16 | CCACTCATCATCAAGCCTAACA | AGGTGGTGCTGGTTGTAGGT |
| ORS-1064 | 16 | TGAATGATCTATGAGTGGTGATGG | ACTCGCAGTGGTAAGTCGTTAGG |
| ORS-495 | 16 | CCAGGATTAGGTAGCTTAGTTCG | GCGATCTGAGGTTGACTCGT |
| ORS-811 | 17 | CCTTCTCCTCAATCTTTGGCTA | AGGAATGAAATGGGTGTGTGT |
| ORS-845 | 17 | GGTGCCCTATCTTCATTCTCTG | CTAAAGGGTATCACACATTTGACATT |
